# 2-(2-Hy­droxy-3-meth­oxy­phen­yl)-1*H*-benzimidazol-3-ium perchlorate

**DOI:** 10.1107/S1600536812023422

**Published:** 2012-05-31

**Authors:** Chuan Chen, Hong-Xing Li, Guang-Feng Hou, Guang-Ming Li

**Affiliations:** a School of Chemistry and Materials Science, Heilongjiang University, Harbin 150080, People’s Republic of China

## Abstract

In the title mol­ecular salt, C_14_H_13_N_2_O_2_^+^·ClO_4_^−^, the ring systems in the cation are almost coplanar [dihedral angle = 5.53 (13)°]. Intra­molecular N—H⋯O and O—H⋯O hydrogen bonds generate *S*(6) and *S*(5) rings, respectively. In the crystal, the two H atoms involved in the intra­molecular hydrogen bonds also participate in inter­molecular links to acceptor O atoms of the perchlorate anions. A simple inter­molecular N—H⋯O bond also occurs. Together, these form a double-chain structure along [101].

## Related literature

For a related structure, see: Yang *et al.* (2010 ▶).
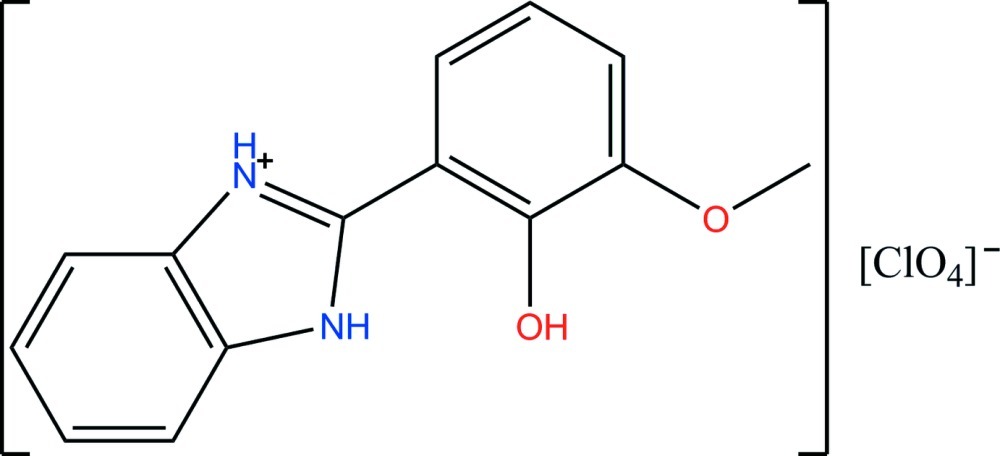


## Experimental

### 

#### Crystal data

C_14_H_13_N_2_O_2_^+^·ClO_4_^−^
*M* *_r_* = 340.71
Monoclinic, 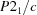

*a* = 7.7698 (16) Å
*b* = 20.462 (4) Å
*c* = 9.856 (2) Å
β = 113.09 (3)°
*V* = 1441.4 (5) Å^3^
*Z* = 4
Mo *K*α radiation
μ = 0.30 mm^−1^
*T* = 293 K
0.50 × 0.45 × 0.42 mm

#### Data collection

Rigaku R-AXIS RAPID diffractometer
Absorption correction: multi-scan (*ABSCOR*; Higashi, 1995 ▶) *T*_min_ = 0.864, *T*_max_ = 0.884
13683 measured reflections
3285 independent reflections
2025 reflections with *I* > 2σ(*I*)
*R*_int_ = 0.061

#### Refinement

*R*[*F* ^2^ > 2σ(*F* ^2^)] = 0.051
*wR*(*F* ^2^) = 0.140
*S* = 1.03
3285 reflections
218 parameters
15 restraints
H atoms treated by a mixture of independent and constrained refinement
Δρ_max_ = 0.37 e Å^−3^
Δρ_min_ = −0.32 e Å^−3^

### 

Data collection: *RAPID-AUTO* (Rigaku, 1998 ▶); cell refinement: *RAPID-AUTO*; data reduction: *CrystalClear* (Rigaku/MSC, 2002 ▶); program(s) used to solve structure: *SHELXS97* (Sheldrick, 2008 ▶); program(s) used to refine structure: *SHELXL97* (Sheldrick, 2008 ▶); molecular graphics: *SHELXTL* (Sheldrick, 2008 ▶); software used to prepare material for publication: *SHELXL97*.

## Supplementary Material

Crystal structure: contains datablock(s) I, global. DOI: 10.1107/S1600536812023422/hb6796sup1.cif

Structure factors: contains datablock(s) I. DOI: 10.1107/S1600536812023422/hb6796Isup2.hkl

Supplementary material file. DOI: 10.1107/S1600536812023422/hb6796Isup3.cml

Additional supplementary materials:  crystallographic information; 3D view; checkCIF report

## Figures and Tables

**Table 1:** Hydrogen-bond geometry (Å, °)

*D*—H⋯*A*	*D*—H	H⋯*A*	*D*⋯*A*	*D*—H⋯*A*
O1—H1⋯O2	0.82 (1)	2.20 (3)	2.635 (3)	114 (3)
O1—H1⋯O4^i^	0.82 (1)	2.13 (2)	2.869 (3)	151 (3)
N1—H101⋯O1	0.90 (1)	2.12 (3)	2.660 (3)	118 (3)
N1—H101⋯O5	0.90 (1)	2.04 (2)	2.819 (3)	144 (3)
N2—H102⋯O3^ii^	0.89 (1)	2.06 (2)	2.857 (4)	149 (3)

Symmetry codes: (i) 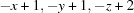; (ii) 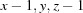.

**Fig. 1.**
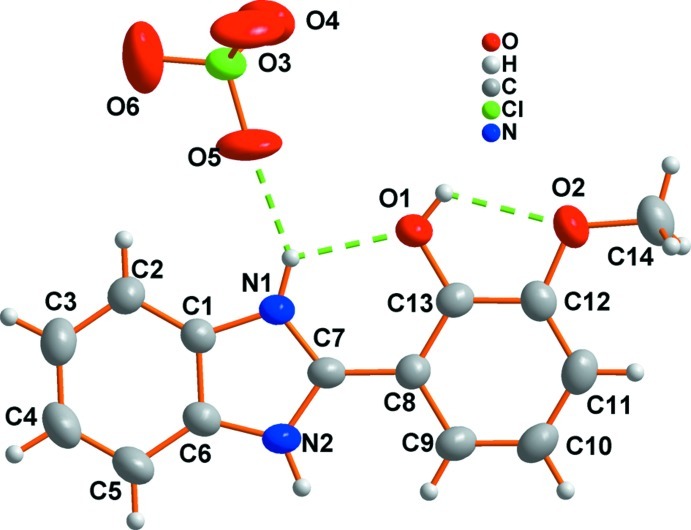
The molecular structure of the title compound, showing 50% probability displacement ellipsoids.

**Fig. 2.**
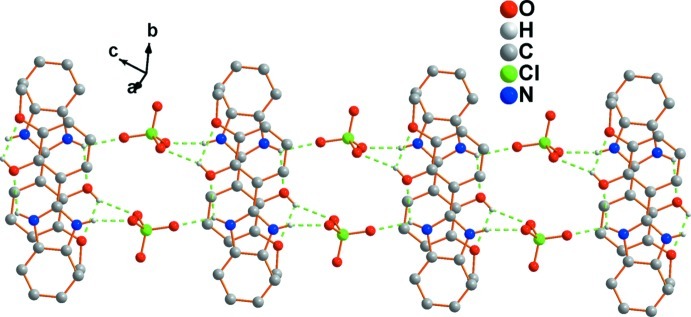
The double chain structure.

## supplementary crystallographic information

### Comment

The title compound is an unexpected product during the process of preparing the N,N'-phenyl-bis(3-methoxysalicylaldimine) and Ce complex. We speculate that the title ligand was produced form the decomposition of N,N'-phenyl-bis(3-methoxysalicylaldimine). A similar ligand and Yb cation constructed metallic cluster has been reproted by Yang's group (Yang *et al.* 2010).

In the title compound, [C_14_H_13_N_2_O_2_][ClO_4_], the phenyl ring and benzimidazol ring are almost conplane with small dihedral angle of 5.517 (1) ° (Figure 1). Intermolecular N—H···O and O—H···0 hydrogen bonds link these protonated ligands and perchlorate anions to form double chain structure along [101] (Figure 2, Table 1).

### Experimental

A solution of N,N'-phenyl-bis(3-methoxysalicylaldimine) (0.1502 g, 0.4 mmol) in 10 mL CH_2_Cl_2_, was added dropwise to a solution of [Ce(ClO_4_)_3_]˙9H_2_O (0.1984 g, 0.4 mmol) in 10 mL of CH_3_OH solution, after then the mixsure was keeping stir about 12h. Pale brown blocks were obtained after seven days. Elemental Anal. Calc. for C_14_H_13_ClN_2_O_6_: C, 49.35; H, 3.85; Cl, 10.41; N, 8.22; O, 28.17 wt%, Found: C, 49.32; H, 3.86; Cl, 10.40; N, 8.24 O, 28.15 wt%.

### Refinement

C-bound H atoms were placed in calculated positions and treated as riding on their parent atoms, with C—H = 0.93 / 0.96 Å (aromatic / methyl) and *U*_iso_(H) = 1.2 \ 1.5*U*_eq_(C). H atoms attached to N and O atoms were located in a differece Fourier map and refined with a restraint of N—H = 0.90 (1) Å and O—H = 0.82 (1), and with *U*_iso_(H) = 1.5*U*_eq_(N and O).

### Crystal data

**Table:**

C_14_H_13_N_2_O_2_^+^·ClO_4_^−^	*F*(000) = 704
*M**_r_* = 340.71	*D*_x_ = 1.570 Mg m^−^^3^
Monoclinic, *P*2_1_/*c*	Mo *K*α radiation, λ = 0.71073 Å
Hall symbol: -P 2ybc	Cell parameters from 7910 reflections
*a* = 7.7698 (16) Å	θ = 3.0–27.4°
*b* = 20.462 (4) Å	µ = 0.30 mm^−^^1^
*c* = 9.856 (2) Å	*T* = 293 K
β = 113.09 (3)°	Block, colorless
*V* = 1441.4 (5) Å^3^	0.50 × 0.45 × 0.42 mm
*Z* = 4	

### Data collection

**Table:**

Rigaku R-AXIS RAPID diffractometer	3285 independent reflections
Radiation source: fine-focus sealed tube	2025 reflections with *I* > 2σ(*I*)
Graphite monochromator	*R*_int_ = 0.061
ω scan	θ_max_ = 27.5°, θ_min_ = 3.0°
Absorption correction: multi-scan (*ABSCOR*; Higashi, 1995)	*h* = −10→9
*T*_min_ = 0.864, *T*_max_ = 0.884	*k* = −26→26
13683 measured reflections	*l* = −12→12

### Refinement

**Table:**

Refinement on *F*^2^	Primary atom site location: structure-invariant direct methods
Least-squares matrix: full	Secondary atom site location: difference Fourier map
*R*[*F*^2^ > 2σ(*F*^2^)] = 0.051	Hydrogen site location: inferred from neighbouring sites
*wR*(*F*^2^) = 0.140	H atoms treated by a mixture of independent and constrained refinement
*S* = 1.03	*w* = 1/[σ^2^(*F*_o_^2^) + (0.0569*P*)^2^ + 0.5284*P*] where *P* = (*F*_o_^2^ + 2*F*_c_^2^)/3
3285 reflections	(Δ/σ)_max_ < 0.001
218 parameters	Δρ_max_ = 0.37 e Å^−^^3^
15 restraints	Δρ_min_ = −0.32 e Å^−^^3^

### Special details

**Table:**

Geometry. All esds (except the esd in the dihedral angle between two l.s. planes) are estimated using the full covariance matrix. The cell esds are taken into account individually in the estimation of esds in distances, angles and torsion angles; correlations between esds in cell parameters are only used when they are defined by crystal symmetry. An approximate (isotropic) treatment of cell esds is used for estimating esds involving l.s. planes.
Refinement. isor 0.01 o5 o6 dfix 0.90 0.01 h101 n1 h102 n2 dfix 0.82 0.01 o1 h1 Refinement of F^2^ against ALL reflections. The weighted R-factor wR and goodness of fit S are based on F^2^, conventional R-factors R are based on F, with F set to zero for negative F^2^. The threshold expression of F^2^ > 2sigma(F^2^) is used only for calculating R-factors(gt) etc. and is not relevant to the choice of reflections for refinement. R-factors based on F^2^ are statistically about twice as large as those based on F, and R- factors based on ALL data will be even larger.

### Fractional atomic coordinates and isotropic or equivalent isotropic displacement parameters (Å^2^)

**Table:**

	*x*	*y*	*z*	*U*_iso_*/*U*_eq_	
O1	0.2919 (3)	0.54938 (10)	0.6641 (2)	0.0448 (5)	
H1	0.318 (5)	0.5726 (15)	0.737 (3)	0.067*	
O2	0.1478 (3)	0.66205 (11)	0.6962 (2)	0.0536 (6)	
O3	0.8344 (4)	0.46040 (16)	0.9925 (3)	0.0933 (10)	
O4	0.5840 (4)	0.41460 (17)	1.0309 (3)	0.0886 (9)	
O5	0.5714 (4)	0.43412 (17)	0.7935 (2)	0.0900 (10)	
O6	0.7608 (6)	0.35259 (16)	0.9385 (4)	0.1203 (13)	
C1	0.3647 (4)	0.39510 (13)	0.4381 (3)	0.0348 (6)	
C2	0.4842 (4)	0.34335 (15)	0.5008 (3)	0.0451 (7)	
H2	0.5525	0.3404	0.6020	0.054*	
C3	0.4962 (4)	0.29644 (16)	0.4046 (4)	0.0528 (8)	
H3	0.5726	0.2603	0.4424	0.063*	
C4	0.3972 (4)	0.30136 (17)	0.2519 (4)	0.0556 (8)	
H4	0.4126	0.2692	0.1910	0.067*	
C5	0.2779 (5)	0.35259 (17)	0.1898 (3)	0.0540 (8)	
H5	0.2119	0.3559	0.0884	0.065*	
C6	0.2609 (4)	0.39922 (14)	0.2863 (3)	0.0401 (6)	
C7	0.1917 (4)	0.48632 (14)	0.3912 (3)	0.0357 (6)	
C8	0.1067 (4)	0.54718 (13)	0.4073 (3)	0.0361 (6)	
C9	−0.0326 (4)	0.57649 (15)	0.2837 (3)	0.0458 (7)	
H9	−0.0722	0.5561	0.1921	0.055*	
C10	−0.1100 (4)	0.63489 (16)	0.2979 (3)	0.0513 (8)	
H10	−0.2025	0.6537	0.2156	0.062*	
C11	−0.0525 (4)	0.66657 (16)	0.4331 (3)	0.0481 (7)	
H11	−0.1035	0.7068	0.4407	0.058*	
C12	0.0804 (4)	0.63794 (14)	0.5558 (3)	0.0412 (7)	
C13	0.1614 (4)	0.57819 (14)	0.5442 (3)	0.0371 (6)	
C14	0.0861 (5)	0.72523 (17)	0.7194 (4)	0.0610 (9)	
H14A	0.1256	0.7573	0.6665	0.091*	
H14B	0.1392	0.7353	0.8228	0.091*	
H14C	−0.0479	0.7255	0.6845	0.091*	
Cl1	0.68517 (11)	0.41471 (4)	0.93839 (7)	0.0463 (2)	
N1	0.3185 (3)	0.45039 (11)	0.4985 (2)	0.0351 (5)	
H101	0.367 (4)	0.4594 (16)	0.5954 (13)	0.053*	
N2	0.1566 (4)	0.45555 (13)	0.2634 (2)	0.0442 (6)	
H102	0.079 (4)	0.4709 (17)	0.177 (2)	0.066*	

### Atomic displacement parameters (Å^2^)

**Table:**

	*U*^11^	*U*^22^	*U*^33^	*U*^12^	*U*^13^	*U*^23^
O1	0.0481 (12)	0.0439 (12)	0.0338 (9)	0.0034 (10)	0.0068 (9)	−0.0052 (8)
O2	0.0597 (14)	0.0452 (13)	0.0546 (12)	0.0044 (11)	0.0210 (11)	−0.0124 (10)
O3	0.0832 (19)	0.114 (3)	0.0514 (14)	−0.0493 (18)	−0.0079 (13)	0.0054 (15)
O4	0.0807 (19)	0.135 (3)	0.0592 (15)	0.0109 (19)	0.0376 (15)	0.0040 (16)
O5	0.0704 (18)	0.139 (3)	0.0349 (12)	−0.0005 (18)	−0.0075 (11)	0.0044 (14)
O6	0.186 (4)	0.062 (2)	0.146 (3)	0.038 (2)	0.100 (3)	0.0105 (19)
C1	0.0364 (14)	0.0327 (15)	0.0362 (12)	−0.0054 (11)	0.0151 (12)	−0.0020 (11)
C2	0.0398 (16)	0.0443 (18)	0.0456 (15)	0.0021 (13)	0.0107 (13)	−0.0011 (13)
C3	0.0423 (17)	0.0423 (19)	0.072 (2)	0.0015 (14)	0.0206 (16)	−0.0054 (15)
C4	0.0515 (19)	0.055 (2)	0.0638 (19)	−0.0042 (16)	0.0265 (17)	−0.0215 (16)
C5	0.058 (2)	0.059 (2)	0.0426 (15)	−0.0010 (17)	0.0173 (15)	−0.0135 (14)
C6	0.0444 (16)	0.0398 (17)	0.0359 (13)	−0.0059 (12)	0.0155 (12)	−0.0038 (11)
C7	0.0386 (15)	0.0365 (15)	0.0295 (12)	−0.0064 (12)	0.0106 (11)	0.0014 (10)
C8	0.0355 (14)	0.0323 (15)	0.0386 (13)	−0.0028 (12)	0.0123 (11)	0.0020 (11)
C9	0.0497 (17)	0.0418 (18)	0.0381 (14)	−0.0027 (14)	0.0089 (13)	0.0052 (12)
C10	0.0509 (19)	0.0452 (19)	0.0512 (17)	0.0056 (15)	0.0129 (15)	0.0152 (14)
C11	0.0478 (18)	0.0363 (17)	0.0629 (18)	0.0025 (14)	0.0245 (16)	0.0070 (14)
C12	0.0423 (16)	0.0353 (16)	0.0492 (15)	−0.0056 (12)	0.0214 (14)	−0.0032 (12)
C13	0.0330 (14)	0.0363 (16)	0.0382 (13)	−0.0067 (12)	0.0100 (11)	0.0022 (11)
C14	0.069 (2)	0.044 (2)	0.075 (2)	−0.0003 (17)	0.0338 (19)	−0.0128 (17)
Cl1	0.0494 (4)	0.0493 (4)	0.0333 (3)	−0.0014 (4)	0.0086 (3)	−0.0021 (3)
N1	0.0388 (12)	0.0355 (13)	0.0277 (10)	−0.0017 (10)	0.0094 (10)	−0.0003 (9)
N2	0.0529 (15)	0.0443 (15)	0.0278 (10)	0.0031 (12)	0.0075 (11)	0.0012 (9)

### Geometric parameters (Å, º)

**Table:**

O1—C13	1.353 (3)	C6—N2	1.376 (4)
O1—H1	0.816 (10)	C7—N2	1.337 (3)
O2—C12	1.365 (3)	C7—N1	1.346 (3)
O2—C14	1.428 (4)	C7—C8	1.447 (4)
O3—Cl1	1.420 (3)	C8—C13	1.398 (4)
O4—Cl1	1.419 (3)	C8—C9	1.408 (4)
O5—Cl1	1.410 (2)	C9—C10	1.369 (4)
O6—Cl1	1.400 (3)	C9—H9	0.9300
C1—C2	1.384 (4)	C10—C11	1.389 (4)
C1—N1	1.389 (3)	C10—H10	0.9300
C1—C6	1.396 (4)	C11—C12	1.375 (4)
C2—C3	1.378 (4)	C11—H11	0.9300
C2—H2	0.9300	C12—C13	1.400 (4)
C3—C4	1.400 (4)	C14—H14A	0.9600
C3—H3	0.9300	C14—H14B	0.9600
C4—C5	1.374 (5)	C14—H14C	0.9600
C4—H4	0.9300	N1—H101	0.897 (10)
C5—C6	1.389 (4)	N2—H102	0.885 (10)
C5—H5	0.9300		
			
C13—O1—H1	111 (3)	C9—C10—H10	119.5
C12—O2—C14	118.0 (3)	C11—C10—H10	119.5
C2—C1—N1	132.2 (3)	C12—C11—C10	119.5 (3)
C2—C1—C6	122.0 (3)	C12—C11—H11	120.2
N1—C1—C6	105.8 (2)	C10—C11—H11	120.2
C3—C2—C1	116.1 (3)	O2—C12—C11	126.5 (3)
C3—C2—H2	121.9	O2—C12—C13	113.1 (3)
C1—C2—H2	121.9	C11—C12—C13	120.5 (3)
C2—C3—C4	122.2 (3)	O1—C13—C8	119.0 (3)
C2—C3—H3	118.9	O1—C13—C12	121.1 (2)
C4—C3—H3	118.9	C8—C13—C12	119.9 (3)
C5—C4—C3	121.6 (3)	O2—C14—H14A	109.5
C5—C4—H4	119.2	O2—C14—H14B	109.5
C3—C4—H4	119.2	H14A—C14—H14B	109.5
C4—C5—C6	116.6 (3)	O2—C14—H14C	109.5
C4—C5—H5	121.7	H14A—C14—H14C	109.5
C6—C5—H5	121.7	H14B—C14—H14C	109.5
N2—C6—C5	132.1 (3)	O6—Cl1—O5	110.6 (2)
N2—C6—C1	106.4 (2)	O6—Cl1—O4	109.7 (2)
C5—C6—C1	121.5 (3)	O5—Cl1—O4	111.48 (18)
N2—C7—N1	107.4 (2)	O6—Cl1—O3	108.5 (2)
N2—C7—C8	125.1 (2)	O5—Cl1—O3	106.87 (18)
N1—C7—C8	127.5 (2)	O4—Cl1—O3	109.64 (19)
C13—C8—C9	118.8 (3)	C7—N1—C1	109.9 (2)
C13—C8—C7	121.2 (2)	C7—N1—H101	127 (2)
C9—C8—C7	120.0 (2)	C1—N1—H101	123 (2)
C10—C9—C8	120.2 (3)	C7—N2—C6	110.5 (2)
C10—C9—H9	119.9	C7—N2—H102	123 (2)
C8—C9—H9	119.9	C6—N2—H102	126 (2)
C9—C10—C11	121.1 (3)		

### Hydrogen-bond geometry (Å, º)

**Table:**

*D*—H···*A*	*D*—H	H···*A*	*D*···*A*	*D*—H···*A*
O1—H1···O2	0.82 (1)	2.20 (3)	2.635 (3)	114 (3)
O1—H1···O4^i^	0.82 (1)	2.13 (2)	2.869 (3)	151 (3)
N1—H101···O1	0.90 (1)	2.12 (3)	2.660 (3)	118 (3)
N1—H101···O5	0.90 (1)	2.04 (2)	2.819 (3)	144 (3)
N2—H102···O3^ii^	0.89 (1)	2.06 (2)	2.857 (4)	149 (3)

Symmetry codes: (i) −*x*+1, −*y*+1, −*z*+2; (ii) *x*−1, *y*, *z*−1.
